# Electrocatalytic Decomposition of Lithium Oxalate-Based Composite Microspheres as a Prelithiation Additive in Lithium-Ion Batteries

**DOI:** 10.3390/molecules29132975

**Published:** 2024-06-22

**Authors:** Jian Liu, Jingyi Lin, Zuwei Yin, Zhen Tong, Junke Liu, Zhen Wang, Yao Zhou, Juntao Li

**Affiliations:** College of Energy, Xiamen University, Xiamen 361102, China; jianl5790@163.com (J.L.); 32420231153112@stu.xmu.edu.cn (J.L.); yinzuwei@xmu.edu.cn (Z.Y.); tongzhen0701@163.com (Z.T.); liujunke555@163.com (J.L.); 32420210156629@stu.xmu.edu.cn (Z.W.)

**Keywords:** lithium-ion battery, prelithiation, lithium oxalate, electrocatalytic, coulombic efficiency, solid electrolyte interface

## Abstract

In conventional lithium-ion batteries (LIBs), the active lithium from the lithium-containing cathode is consumed by the formation of a solid electrolyte interface (SEI) at the anode during the first charge, resulting in irreversible capacity loss. Prelithiation additives can provide additional active lithium to effectively compensate for lithium loss. Lithium oxalate is regarded as a promising ideal cathode prelithiation agent; however, the electrochemical decomposition of lithium oxalate is challenging. In this work, a hollow and porous composite microsphere was prepared using a mixture of lithium oxalate, Ketjen Black and transition metal oxide catalyst, and the formulation was optimized. Owing to the compositional and structural merits, the decomposition voltage of lithium oxalate in the microsphere was reduced to 3.93 V; when being used as an additive, there is no noticeable side effect on the performance of the cathode material. With 4.2% of such an additive, the first discharge capacity of the LiFePO4‖graphite full cell increases from 139.1 to 151.9 mAh g^−1^, and the coulombic efficiency increases from 88.1% to 96.3%; it also facilitates the formation of a superior SEI, leading to enhanced cycling stability. This work provides an optimized formula for developing an efficient prelithiation agent for LIBs.

## 1. Introduction

Lithium-ion batteries have become an indispensable power source for various portable devices and electric vehicles due to their high working voltage, long cycle life, safety and environment-friendliness [1,2,3,4,5]. However, with the increasing demand for longer battery life and higher energy density, it is essential to improve the energy density of lithium-ion batteries [6,7,8,9,10]. Although various high-specific-capacity energy anode materials have been extensively studied [11,12,13,14], the generation of SEI during the first charge of lithium-ion batteries results in irreversible lithium loss, leading to a decrease in energy density [15,16,17,18,19]. To resolve this issue, prelithiation has emerged as an effective strategy. Among the various prelithiation methods reported, the prelithiation of cathode additives offers the advantages of a simple process, low price and high safety, making it a promising application prospect [9,20]. To date, cathode prelithiation additives such as Li_2_O [21], Li_2_O_2_ [22], Li_3_N [23], Li_2_NiO_2_ [24], Li_5_FeO_4_ [25] and some lithium-rich composites [26,27,28,29] have been reported. However, these additives are generally sensitive to air humidity and may produce oxygen during decomposition, which can cause damage to the battery system; moreover, lithium-rich composites might be too expensive to utilize on a large scale. As such, further research is needed to develop cathode prelithiation additives that are both cost-effective and environmentally stable.

Lithium oxalate has been proposed as a sacrificial salt [30]. During charging, only Li^+^ and CO_2_ are generated, while the gas produced can be extracted by vacuum from the battery system before sealing during the formation process [31]. Because of its theoretical capacity of 545 mAh·g^−1^, low cost, remarkable air stability and its lack of decomposition residue, it is an ideal cathode prelithiation additive. However, it suffers from poor conductivity and high decomposition voltage (4.7 V vs. Li/Li^+^ [32], much higher than the theoretical decomposition voltage of 3 V vs. Li/Li^+^ [33]). A high operating voltage can easily lead to a rapid degradation of battery performance [34]. The current research on using lithium oxalate as a supplement additive has primarily focused on reducing the decomposition voltage by incorporating catalysts [35,36,37,38]. The way that lithium oxalate solid particles are formulated with the conductive agent and the catalyst solids can affect their decomposition voltage significantly; the mass fraction of each component can therefore also be optimized to maximize the fraction of lithium oxalate, which meanwhile does not compromise its decomposition.

In this work, we have attempted to minimize the fraction of the conductive agent and meanwhile to optimize the interfacial contact among the different solids for the efficient electrocatalytic decomposition of lithium oxalate. The homogeneous mixture of lithium oxalate, the conductive carbon and the transition metal catalyst were modulated into porous hollow microspheres, and the complete decomposition of lithium oxalate was achieved at 3.93 V vs. Li/Li^+^; the voids from the hollow structure of the microspheres help to mitigate the impact of gas produced from the decomposition of lithium oxalate on the cathode active material, eliminating the negative effect on the long-term cycling. When introduced into the LiFePO_4_‖graphite battery, the coulombic efficiency increases from 88.1% to 96.3%; a thinner Li_2_CO_3_-rich inorganic SEI was also observed. These together improve the cycling stability, proving the potential of this innovative approach to enhance the performance of lithium-ion batteries.

## 2. Results

### 2.1. Recrystallization and Optimization of the Conductive Agent

**Figure 1:**
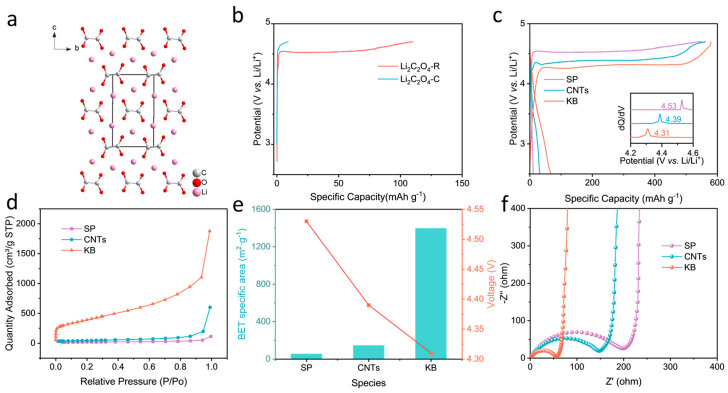
(**a**) The crystal structure of Li_2_C_2_O_4_. (**b**) Charge curve of commercial lithium oxalate (Li_2_C_2_O_4_−C) and recrystallized lithium oxalate (Li_2_C_2_O_4_−R) with a mass ratio of 8:1:1 using super p as the conductive agent at 0.05 C. (**c**) First cycle charge–discharge curves of Li_2_C_2_O_4_−R using different conductive agents with a mass ratio of 6:3:1 at 0.05 C (inset is corresponding dQ/dV curves). (**d**) BET measurement curves of SP, CNTs and KB. (**e**) Relationship between decomposition voltage and BET of the conductive carbon. (**f**) Electrochemical impedance spectroscopy of lithium oxalate with different conductive agents.

Li_2_C_2_O_4_ consists of an oxalate anion riveted with two lithium ions (Figure 1a). Commercial Li_2_C_2_O_4_ was analysed first. Thermogravimetric analysis (TGA) reveals a decomposition temperature close to 500 °C (Figure S1), indicating its high thermal stability. The commercial Li_2_C_2_O_4_ particles were observed to be larger than tens of microns in size using field emission scanning electron microscopy (SEM) (Figure S2); such a large size would be disadvantageous when homogeneously mixing them with the other solid components to be used as a prelithiation additive, and it is also not favourable for the electrochemical activity, considering the difficulty of charge transfer in the large insulating microsphere. Hence, reducing the particle size is necessary. As the solubility of Li_2_C_2_O_4_ in water varies more widely than that in ethanol, as it is 5.87 g per 100 g of water at 25 °C and almost impossible in ethanol [39], respectively, this provides a chance to manipulate its size through recrystallization. Figure S3 shows that both commercial lithium oxalate (Li_2_C_2_O_4_-C) and recrystallized lithium oxalate (Li_2_C_2_O_4_-R) exhibited typical X-ray diffraction (XRD) peaks of lithium oxalate, indicating that no impurities were introduced through recrystallization. importantly, the particle size of recrystallized Li_2_C_2_O_4_-R was reduced to less than 10 μm (Figure S4).

Their electrochemical decomposition behaviours at 0.05 C between 2.5 and 4.7 V were further compared. Figure 1b shows that, when traditional super p is used as the conductive agent and the ratio of active material/conductive agent/binder is 8:1:1, the capacity of the commercial lithium oxalate is 9.1 mAh·g^−1^, which is much lower than its theoretical value during the first charge. It has been much improved after recrystallization, yet the capacity is 110.3 mAh·g^−1^, which is still lower than expected. When improving the fraction of the conductive agent, that is, with the ratio of lithium oxalate/conductive agent/binder as 6:3:1, the first charge capacity is 551.6 mAh·g^−1^ when being charged to 4.7 V, and the first discharge capacity is 12.2 mAh·g^−1^ (Figure 1c); in the subsequent charge and discharge cycles, no significant capacity contribution was observed (Figure S5), indicating that increasing the mass fraction of the conductive agent results in complete delithiation during the first charging process, which is important for a suitable prelithiation agent. However, it can be seen that the average decomposition voltage of lithium oxalate exceeds 4.5 V, which is beyond the stable operating voltage range of most cathode materials and commercial electrolytes. Therefore, it is necessary to reduce the decomposition voltage for practical application. To this end, the conductive agent was optimized first. Three types of conductive carbons, namely the super p, Ketjen Black (KB) and carbon nanotubes, were compared. When the carbon nanotubes were used as the conductive agent, the decomposition potential was reduced to 4.39 V, whereas for KB it was decreased to 4.31 V. After charging, the typical XRD peaks of Li_2_C_2_O_4_ completely disappeared, proving that Li_2_C_2_O_4_ was completely decomposed (Figure S6). Figure 1d illustrates the specific surface areas of the three conductive agents: KB with 1397.3 m^2^ g^−1^, CNTs with 146.3 m^2^ g^−1^ and SP with 54.9 m^2^ g^−1^. As the specific surface area of the conductive additive increases, the decomposition voltage of Li_2_C_2_O_4_ gradually decreases (Figure 1e). The three conductive agents are shown in Figure S7, with KB showing a smaller particle size that allows for more intimate contact with lithium oxalate particles, resulting in a tighter conductive network. Further, in Figure 1f, the comparison of the relevant electrochemical impedance spectroscopy demonstrates that super p has the highest charge transfer impedance before charging, followed by CNTs, with KB exhibiting the smallest. These results together emphasize that intimate interfacial contact between the lithium oxalate and the conductive carbon is crucial to reduce the lithium oxalate’s decomposition voltage.

### 2.2. Characterization and Formulation Optimization of the Lithium Oxalate-Based Composite Microsphere as a Prelithiation Agent

**Figure 2:**
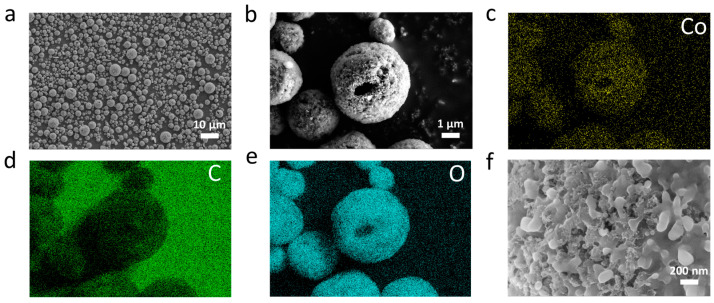
(**a**,**b**,**f**) SEM images of LCK80 at different magnifications. EDS patterns of (**c**) Co, (**d**) C and (**e**) O in LCK80.

Though KB was identified as the optimal conductive agent, reducing the peak oxidation voltage of lithium oxalate to 4.31 V, the utilization of excessive conductive agents remains an issue. Moreover, when used a prelithiation agent, it needs to be mixed with the cathode active materials, which would impact the contact between lithium oxalate and the conductive agent. To address these issues, we introduced Co_3_O_4_ as an electrocatalyst, mixed with KB and lithium oxalate in water to make a slurry for the preparation of secondary composite microspheres (which are named LCK*x*, where L denotes lithium oxalate added, C denotes Co_3_O_4_ added, K denotes KB added and *x* denotes the percentage content of lithium oxalate) through spray drying. As presented in Figure 2a, the thus-obtained microspheres are spherical with particle sizes less than 10 μm; as is further shown in Figure 2b, cavities can be observed in the microspheres, indicating that they are hollow and porous. From the elemental distribution of the composite microspheres, it can be seen that C, O and Co elements are uniformly distributed on the surface of the particles, indicating that lithium oxalate, KB and Co_3_O_4_ are evenly mixed (Figure 2c–e). In the shell of the microspheres, lithium oxalate is evenly wrapped on the surface of KB and Co_3_O_4_ nanoparticles, indicating intimate contact among the three solid components (Figure 2f).

**Figure 3:**
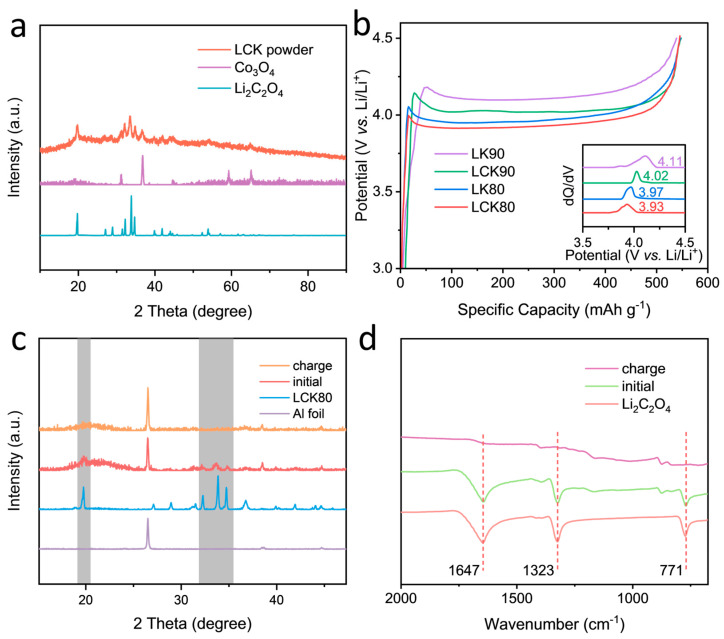
(**a**) XRD patterns of LCK80 and its synthetic raw materials Co_3_O_4_ and Li_2_C_2_O_4_. (**b**) Decomposition curves of different ratios of KB and Co_3_O_4_ at 0.05 C (inset is corresponding dQ/dV curves). (**c**) XRD pattern of the LCK80 electrode before and after charging. (**d**) FT−IR spectra of the LCK80 electrode before and after charging.

In Figure 3a, the composite microspheres showed characteristic peaks corresponding to lithium oxalate and Co_3_O_4_, with broadened peak shapes indicating reduced material size and relatively weak crystallinity, which was due to the fact that the scale of lithium oxalate was greatly reduced after spray drying. The result indicates that the properties of lithium oxalate and Co_3_O_4_ remain unchanged after the formation of composite microspheres.

The electrochemical behaviours of these composite microspheres with varying ratios of Co_3_O_4_ and KB were explored. In order to compare the effects of Co_3_O_4_ and KB, microspheres containing only lithium oxalate and KB without catalyst were synthesized and named LK*x*. Figure 3b reveals that, when only 10% KB is used and no Co_3_O_4_ is added (LK90), the decomposition voltage of lithium oxalate is found to be 4.11 V. This clearly indicates that modulating the mixture of the conductive agent and lithium oxalate into microspheres by spray drying is more effective than simply mixing them. The introduction of Co_3_O_4_ can further reduce the decomposition voltage even when only 5% Co_3_O_4_ and 5% KB are added (LCK90), and the decomposition voltage of lithium oxalate decreases with increasing Co_3_O_4_ and KB. For instance, the decomposition voltage of lithium oxalate is 3.93 V when adding 10% Co_3_O_4_ and KB (LCK80), lower than that when 20% KB is introduced (LK80). However, once the total mass fraction of Co_3_O_4_ and KB reaches 30%, the actual capacity of the composite will be less than 400 mAh g^−1^, which would fail to meet the capacity requirement for a typical cathode prelithiation agent [26]. It is worth mentioning that adding an oxide catalyst is more efficient than simply adding a conductive agent, as it can help maximize the mass fraction of lithium oxalate and meanwhile does not compromise the decomposition voltage. To evaluate the catalytic effect of Co_3_O_4_ on the electrolyte, a Co_3_O_4_‖Li half cell was assembled. As shown in Figure S8, though the decomposition of the carbonate electrolyte also occurred with the presence of a metal catalyst, however, it was almost negligible compared to the capacity delivered by the prelithiation agent. By comparing the XRD pattern (Figure 3c) and FI-IR spectra (Figure 3d) of the LCK80 electrode before and after charging, it can be found that the XRD characteristic peaks and infrared absorption peaks characteristic for lithium oxalate disappeared after charging, indicating that it has been irreversibly decomposed.

**Figure 4:**
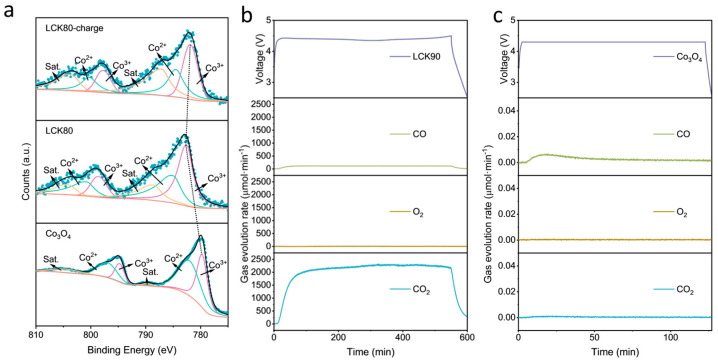
(**a**) Co 2p XPS of Co_3_O_4_, the pristine and charged LCK80. (**b**) DEMS curves of LCK90 and (**c**) Co_3_O_4_.

Based on specific capacity and decomposition voltage, the two samples, LCK90 and LCK80, were further characterized. Figure 4a shows the Co 2p XPS spectra of the original Co_3_O_4_, and the pristine as well as the charged LCK80 electrode. The binding energy for the lower oxidative species Co^3+^ in Co 2p_3/2_ shifts from 779.7 eV to 782.7 eV after sand grinding and spray drying, indicating the oxidation or the interfacial interaction between the oxide catalyst and the lithium oxalate. After charging, the binding energy of Co^3+^ in Co 2p_3/2_ shifts slightly from 782.7 eV to 781.9 eV, indicating that Co_3_O_4_ participates in the transfer of electrons during the charging process.

The decomposition process of LCK90 was tracked by following the various gases (including CO_2_, O_2_ and CO) produced during charging using differential electrochemical mass spectrometry (DEMS, Figure 4b). Note that the decomposition voltage for the above DEMS analysis was a bit higher than that measured in Figure 4b, which is because a carbon paper current collector was employed rather than aluminium foil. The results showed that the material produces a large amount of carbon dioxide and trace amounts of carbon monoxide during charging, confirming the oxidation of the oxalate groups. At the same time, a similar test at a constant voltage of 4.3 V was carried out on the electrode which was prepared using only Co_3_O_4_ (i.e., no lithium oxalate). The results depicted in Figure 4c reveal that only a tiny amount of CO was produced, and the level was similar to that of LCK90. Considering that an oxidation reaction occurs during the charging process of Li_2_C_2_O_4_, the valence state of the carbon element should change from +3 to +4, that is, from oxalate to CO_2_. Therefore, Li_2_C_2_O_4_ releases Li^+^ when charged and the gas decomposition product is only CO_2_. It can be determined that the reaction equation of Li_2_C_2_O_4_ during charging is as follows:

Li_2_C_2_O_4_ − 2e^−^ → 2Li^+^ + 2CO_2_ (1)

### 2.3. Application of the Lithium Oxalate-Based Composite Microsphere as a Prelithiation Agent

**Figure 5:**
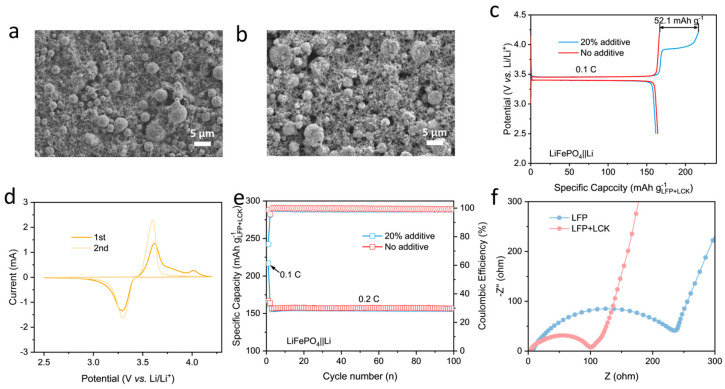
Morphology of the (**a**) pristine and (**b**) charged LCK80. (**c**) Charge−discharge curves of LiFePO_4_ cathode with and without LCK80 in half cells; (**d**) the first 2 CV curves of LiFePO_4_ cathode with LCK80. (**e**) Cycling performance LiFePO_4_ cathode with and without LCK80 in half cells; (**f**) EIS spectra of the pristine electrodes.

The electrode of LCK80 before and after cycling was characterized by SEM observation (Figure 5a,b). It can be seen that the LCK80 microspheres maintain good structural integrity in the electrode; after charging, the microspheres become quite fractured, owing to the decomposition of the lithium oxalate, which is well dispersed within the wall of the microsphere. It should also be mentioned that the hollow and porous structural features of the composite microspheres provide channels to release the gas produced during the charging process, which thus alleviates its impact on the structural stability of the electrode. The performance of the LCK as a prelithiation agent was further evaluated in LiFePO_4_‖Li half cells. Excess LCK80 (20%) was mixed with LiFePO_4_ to prepare a composite electrode; as shown in Figure 5c, compared to the LiFePO_4_ electrode with no LCK80, an additional charging platform between 3.9 and 4 V appeared in the first charging profile. When normalized by the total weight of the LiFePO_4_ and LCK80, the presence of the LCK80 leads to an increase of 53.2 mAh g^−1^, theoretically. The first charge capacity of LiFePO_4_ is 166.6 mAh g^−1^ and the capacity is 218.7 mAh g^−1^ with 20% LCK80 introduced, so the actual increase is 52.1 mAh g^−1^. Such a result proves the remarkable compatibility of LCK in the LiFePO_4_-based electrode. Figure 5d displays the first two CV curves of the composite electrode. It exhibits a new oxidation peak around 4.0 V in the first cycle, which disappears in the second cycle, showing that the decomposition is irreversible. Accordingly, as shown in Figure 5e, in the first cycle, owing to the irreversible decomposition of LCK80, the coulombic efficiency of the composite electrode is lower than the one with no prelithiation agent. However, in the subsequent cycling process no significant attenuation of capacity or efficiency is observed (Figure 5e), indicating no side effects from the presence of LCK80 on the electrode cycling performance. The hybrid electrode of LiFePO_4_ and LCK80 before and after charging was characterized by SEM observation (Figure S9a,b). It can be observed that the hybrid electrode remains structurally intact before and after charging, indicating that the introduction of excess LCK80 will not cause significant damage to the electrodes due to the gas generated during the charging process. Moreover, as shown in Figure 5d, EIS analysis prior to charging shows that the addition of the LCK80 can decrease the impedance of the electrode, suggesting the excellent conductivity of LCK80 (Figure 5f). These results prove the workability of LCK as a prelithiation agent in a LiFePO_4_-based electrode.

The influence of LCK80 on the performance of the LiFePO_4_‖graphite full cell was investigated in further detail. The amount of LCK80 required can be determined by Equation (2), where *M* represents the amount needed, *C_c_* represents the theoretical specific capacity of the positive electrode, *R* represents the N/P ratio, *CE_n_* represents the initial coulombic efficiency of the negative electrode and *C_p_* represents the theoretical specific capacity of the additive, as follows:

(2) $$M=\frac{Cc\times R\times (1-CEn)}{Cp}$$

**Figure 6:**
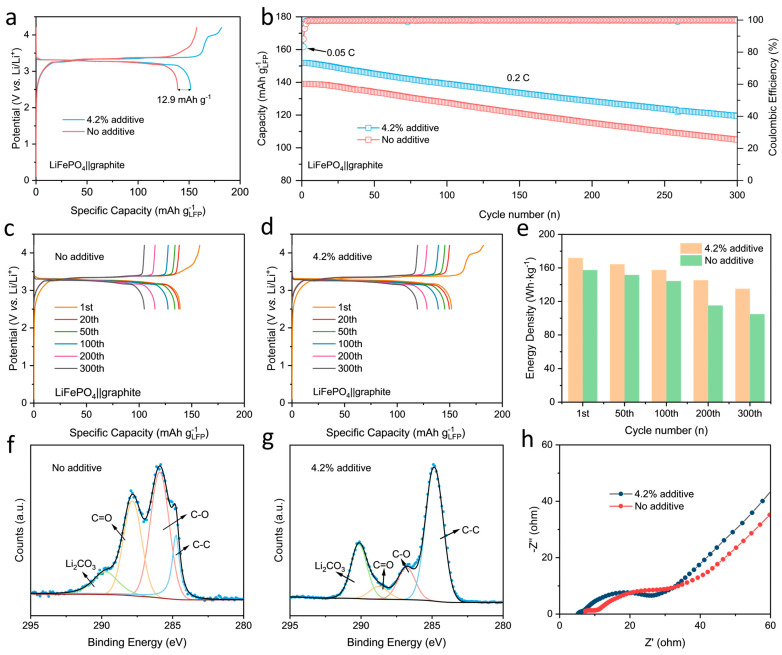
(**a**) The first charge−discharge curves and (**b**) cycling performance of the LiFePO_4_‖graphite full cell; charge and discharge curves of the 1st, 20th, 50th, 100th, 200th and 300th cycles of the LiFePO_4_‖graphite full cell (**c**) without and (**d**) with additive; (**e**) comparison of energy density of full cells in different cycles. C 1 s XPS spectra of graphite after the first cycle of full cell (**f**) without and (**g**) with additive; (**h**) electrochemical impedance spectroscopy after the first cycle of the full cells.

As shown in Figure S10a, the initial coulombic efficiency of graphite is 91%, assuming N/P = 1.2, and the required LCK80 addition can be calculated from Equation (2) to be 4.2%. On this basis, 4.2% of the additive was introduced into the cathode; a control electrode was prepared with no lithiation additive. The first cycle charge–discharge curve of the full cell at a rate of 0.05 C is shown in Figure 6a. For the electrode with LCK, an additional platform was observed after the LiFePO_4_ charging platform, owing to the electrochemical decomposition of the lithium oxalate. The charging capacity for the cell with LCK was 182.3 mAh g^−1^, and the discharge capacity was 151.9 mAh g^−1^. In comparison, the control cell delivered a charging capacity of 157.8 mAh g^−1^ and discharging capacity of 139.1 mAh g^−1^. That is, the discharge capacity increased by 12.9 mAh g^−1^ owing to the presence of LCK, transforming into 9.3% of the capacity of the control cell. When the cathode is calculated only for LiFePO_4_, the coulombic efficiency increases from 88.1% to 96.3%. As is further shown in Figure 6b, the cycling performance of both full cells at 0.2 C is similar, showing that the presence of the prelithiation additive has no side effect on the long-term stability. The charge–discharge curves of the full cells for different cycles within 300 cycles are compared in Figure 6c,d; they further prove the increased reversible capacity and stable cycling performance for the cell with the LCK prelithiation additive. Such results clearly justify the role of the LCK in replenishing the active lithium loss from the initial cycle.

The energy density of the full cells in different cycles is also compared. The cell with the additive displayed higher energy density than the control during cycling. After prelithiation, the energy density of the battery increased from 157.4 Wh·kg^−1^ to 171.9 Wh·kg^−1^ in the first cycle. The energy density of control group decayed more rapidly during the cycling process, reducing from 144.4 Wh·kg^−1^ in the 100th cycle to 115.2 Wh·kg^−1^ in the 200th (Figure 6e). The C 1s XPS spectra of the graphite anode in both cells after the first cycle are shown in Figure 6f and Figure 6g, respectively. The intensity of the C-O and C=O peaks for the control anode was stronger than that with additives, indicating that the SEI formed in the control cell contains more organic components. In comparison, for the cell with the prelithiation additive, a stronger intensity of C-C and Li_2_CO_3_ was observed; considering that XPS is a surface technique, such a result indicates that the SEI in the cell with the lithiation additive is thinner and rich in Li_2_CO_3_. Inorganic components tend to have higher ionic conductivity and thermal stability compared to the organic components of SEI, leading to better kinetics and cycling stability [40,41]. Figure 6h compares the impedance of the full cells after the first cycle; consistently, the additive-free battery exhibits higher SEI impedance and charge transfer impedance. The kinetics of the cathode and anode were further evaluated by EIS, and the results are shown in Figure S11. After the first charge, no significant difference in the charge transfer impedance of the LFP cathode in the full cells was observed, but the graphite anode showed lower SEI impedance and charge transfer impedance, indicating that the kinetics of the anode were improved. In addition, the effect of prelithiation additives on the rate performance was evaluated, and the results are shown in Figure S12. The full cell with the additives introduced has a higher capacity at different rates, and the difference is more pronounced at higher rates. On the one hand, the introduction of additives reduces the electrochemical impedance and improves the kinetics. On the other hand, the prelithiation additives have a porous structure, which can provide more pathways for Li^+^ in the electrode, which is conducive to the rapid transport of Li^+^ from the electrode to the electrolyte. These factors have led to the improvement of the rate performance.

## 3. Materials and Methods

### 3.1. Materials

Lithium oxalate (99%) was purchased from Shanghai Acmec Biochemical Technology Co., Ltd. (Shanghai, China). Co_3_O_4_ (99.99%) was obtained from Shanghai Aladdin Biochemical Technology Co., Ltd. (Shanghai, China). Carbon nanotubes (CNTs) were purchased from the Nanjing XFNANO Materials Tech Co., Ltd. (Nanjing, China). Electrolytes were purchased from Suzhou Dodochem Co., Ltd. (Suzhou, China). Super P, Ketjen black (EC 600-JD), polyvinylidene difluoride (PVDF), N-methyl-2-pyrrolidone (NMP), LiFePO_4_ and graphite were obtained from Guangdong Canrd New Energy Technology Co., Ltd. (Guangzhou, China). All materials were used as received without further modification.

### 3.2. Recrystallization of Li_2_C_2_O_4_

Briefly, 1 g of commercial Li_2_C_2_O_4_ was dissolved in 50 mL of deionized water and added dropwise to 100 mL of ethanol at a rate of 10 drops per minute. After agitation for 10 min, the resulting solution was filtered and washed three times with ethanol. The obtained material was dried at 200 °C for 4 h to obtain the recrystallized Li_2_C_2_O_4_.

### 3.3. Synthesis of the Lithium Oxalate-Based Composite Microsphere

Lithium oxalate, Ketjen black (KB) and Co_3_O_4_ with different mass ratios were dispersed in water (the total solid content was 1%) and sand-ground first. In the conducted experiments, the ratio of Co_3_O_4_ and KB was maintained at 1:1. To simplify the naming convention of the composites used in the experiments, they were abbreviated using a combination of the initials of their constituent materials along with the percentage content of lithium oxalate. For instance, LCK80 represents a composite consisting of 80% lithium oxalate and 10% Co_3_O_4_ along with 10% KB, while LK90 denotes a composite containing 90% lithium oxalate and 10% KB. Specifically, in the case of LCK80, a mixture of 4 g lithium oxalate, 0.5 g Co_3_O_4_ and 0.5 g KB was blended with 500 mL of deionized water and vigorously stirred at 2000 rpm for 10 h to form a homogeneous dispersion. Subsequently, the solution was spray-dried at a rate of 20 mL per minute at 240 °C to obtain the composite microsphere, which was used as the prelithiation additive.

### 3.4. Material Characterization

Thermal decomposition of Li_2_C_2_O_4_ was investigated using a STA449F5 thermogravimetric analyser (Netzsch, Bavaria, Germany) at a heating rate of 10 °C min^−1^ under nitrogen flow from room temperature to 600 °C. X-ray diffraction (XRD) measurements were performed on a Rigaku Ultima IV X-ray diffractometer with Cu Kα radiation. Field emission scanning electron microscopy (SEM) was conducted using a GeminiSEM 500 microscope from Zeiss. Fourier-transform infrared spectroscopy (FT-IR) tests were performed on an iN10 (Thermo Fisher, Waltham, MA, USA) spectrometer. Nitrogen sorption isotherms were measured with a Micromeritics ASAP 2000 analyser. Specific surface areas were calculated using the Brunauer–Emmet–Teller method. X-ray photoelectron spectroscopy (XPS) was conducted with ESCALAB Xi+ (Thermo Fischer). The tests for the Differential Electrochemical Mass Spectrometer (DEMS) were undertaken by QAS100Li (Linglu Shanghai, China), and the electrodes were prepared by applying a slurry to carbon paper and then assembling them into a Swagelok battery. The loading values of LCK90 and pure Co_3_O_4_ electrodes were 22.4 mg and 11.2 mg, respectively. For the LCK90 electrode the charge current was 380 µA, while it was 190 µA for pure Co_3_O_4_ electrode.

### 3.5. Electrochemical Characterization

Electrochemical performances were evaluated with CR2025 coin cells. The cells were assembled in an argon-filled glove box using 1 M LiPF_6_ in a mixture of ethylene carbonate (EC), dimethyl carbonate (DMC) and diethyl carbonate (DEC) (1:1:1 vol%) as the electrolyte and a Celgard 2300 membrane as the separator. The slurry coating for electrode fabrication was carried out at ambient conditions by mixing the active materials, carbon black and polyvinylidene fluoride (PVDF) binder in N-methyl-2-pyrrolidone (NMP) solvent. The electrodes were then dried in vacuum at 100 °C for 10 h.

The following electrodes were fabricated: (1) The pure Li_2_C_2_O_4_ electrode consisted of 60% Li_2_C_2_O_4_, 30% conductive carbon and 10% PVDF binder in weight, with a typical loading of ~1 mg cm^−2^. (2) The prelithiation additive cathode consisted of 80% as-prepared prelithiation additive, 10% Super P and 10% PVDF binder in weight, with a typical loading of ~1 mg cm^−2^. (3) For preparation of the LiFePO_4_ cathode, the LiFePO_4_ was first mixed with the prelithiation additive with a weight ratio of 20% in half cells and 4.2% in full cells; for half cells, LiFePO_4_ electrodes were prepared with 90% of the aforementioned mixture, 10% carbon black and 10% PVDF, typically with a total loading of ~6 mg cm^−2^; for full cell, LiFePO_4_ electrodes were prepared with 93% of these hybrid cathode materials, 4% carbon black and 3% PVDF, with a typical mass loading of ~11.4 mg cm^−2^. (4) Graphite electrodes were made by mixing the graphite powder, Super P, CMC and SBR at a mass ratio of 96:1:1.2:1.3, and the loading of the active material of the anode was ~5.7 mg cm^−2^. The capacity ratio of the anode to the cathode (N/P ratio) used for the full cell test was 1.1.

The galvanostatic charge/discharge measurement was carried out in the cutoff potential range 2.5–4.2 V. The current density for LiFePO_4_ electrodes is 0.05 C for the first cycle and 0.2 C for the following cycles in the full cell. The assembled cells were galvanostatically cycled at room temperature using a program-controlled test system (LAND CT2001A). Specific capacity was calculated based on the mass of the cathode active material unless it was specified. Cyclic voltammetry (CV) measurements were carried out on a CHI 660E instruments testing system. Electrochemical impedance spectroscopy (EIS) experiments were conducted in the frequency range of 0.01–100,000 Hz by applying an AC voltage of 5 mV amplitude.

## 4. Conclusions

In summary, we have successfully prepared porous hollow composite microspheres as an efficient prelithiation additive using Li_2_C_2_O_4_ as the lithium source, Co_3_O_4_ as the catalyst and a conductive agent through sand grinding and spray drying. Ketjen Black was identified as the optimal conductive agent compared to Super P or carbon nanotubes; the composition of each component was also optimized. Owing to the structural and compositional merits, the Li_2_C_2_O_4_ in the composite microspheres can be electrochemically decomposed at a voltage as low as 3.93 V, and an obvious increase in the discharge capacity can be observed. Additionally, the gas produced during the decomposition of Li_2_C_2_O_4_ can be channelled out due to the microsphere’s hollow and porous structure, and thus it has little impact on the cathode’s cycling performance. These, together with lithium oxalate-based composite microspheres, make an excellent cathode prelithiation additive. With the presence of 4.2% of such an additive in the LiFePO_4_‖graphite full cell, the coulombic efficiency increases from 88.1% to 96.3%. Furthermore, the anode forms a superior SEI during the cycling process, further showcasing the effectiveness of this additive.

## Data Availability

Data are contained within the article or Supplementary Materials.

## Supplementary Materials

The following supporting information can be downloaded at: https://www.mdpi.com/article/10.3390/molecules29132975/s1, Figure S1: Thermogravimetric curve of commercial Li_2_C_2_O_4_ (Li_2_C_2_O_4_-C); Figure S2: SEM image of commercial lithium oxalate (Li_2_C_2_O_4_-C); Figure S3: XRD patterns of commercial lithium oxalate (Li_2_C_2_O_4_-C) and recrystallized lithium oxalate (Li_2_C_2_O_4_-R); Figure S4: SEM image of recrystallized lithium oxalate (Li_2_C_2_O_4_-R); Figure S5: Charge–discharge curves of Li_2_C_2_O_4_-R from the 2nd to 5th cycles; Figure S6: XRD patterns of Li_2_C_2_O_4_ electrodes using different conductive agents before and after charging: (a) SP, (b) CNTs and (c) KB; Figure S7: SEM images of (**a**) SP, (**b**) CNTs and (**c**) KB; Figure S8: The five charge–discharge curves of Co_3_O_4_‖Li half cell; Figure S9: SEM images of the hybrid electrode of LiFePO4 and LCK80 (**a**) before and (**b**) after charging; Figure S10: (**a**) Discharge–charge curve of graphite at first cycle at 0.1 C. (**b**) Cycling performance of graphite at 0.2 C (1 C = 372 mAh g^−1^). Figure S11: Electrochemical impedance spectroscopy of (a) LFP, (b) graphite after the first cycle; Figure S12: Rate performance of LFP‖graphite full cells.
