# Supplementary material for: Electrocatalytic Decomposition of Lithium Oxalate-Based Composite Microspheres as a Prelithiation Additive in Lithium-Ion Batteries
Source: Molecules. 2024 Jun 22;29(13):2975. doi: 10.3390/molecules29132975 (PMC11243468; doi:10.3390/molecules29132975)
Supplement: Supplementary file 1 [file molecules-29-02975-s001.zip › molecules-3039073-supplementary.pdf]

molecules

Supporting Information

Electrocatalytic decomposition of lithium-oxalate-based composite microspheres as prelithiation additive in lithium-ion batteries

Jian Liu [1], Jingyi Lin [1], Zuwei Yin [1], Zhen Tong [1], Junke Liu [1], Zhen Wang [1], Yao Zhou [1,*] and Juntao Li [1,*]

[1] College of Energy, Xiamen University, Xiamen 361102, China;jianl5790@163.com (J.L., Jian Liu); 32420231153112@stu.xmu.edu.cn (J.L., Jingyi Lin); yinzuwei@xmu.edu.cn (Z.Y.); tongzhen0701@163.com (Z.T.); liujunke555@163.com (J.L., Junke Liu); 32420210156629@stu.xmu.edu.cn (Z.W).

* Correspondence: zhouy@xmu.edu.cn (Y.Z.); jtli@xmu.edu.cn (J.L., Jun-Tao Li)

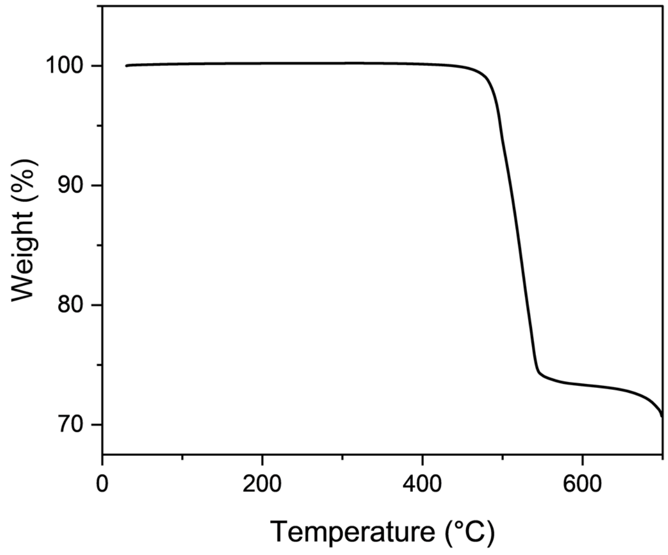

Figure S1. Thermogravimetric curve of commercial $Li_2C_2O_4$ ($Li_2C_2O_4$-C).

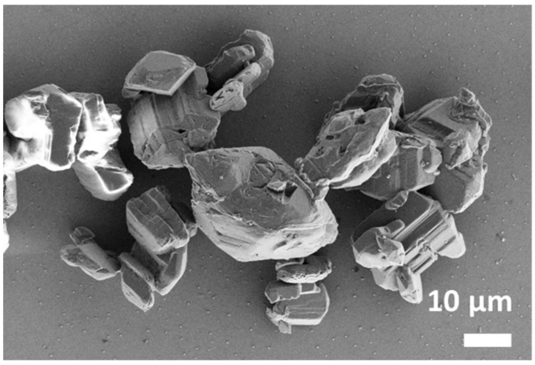

Figure S2. SEM image of commercial lithium oxalate ($Li_2C_2O_4$-C).

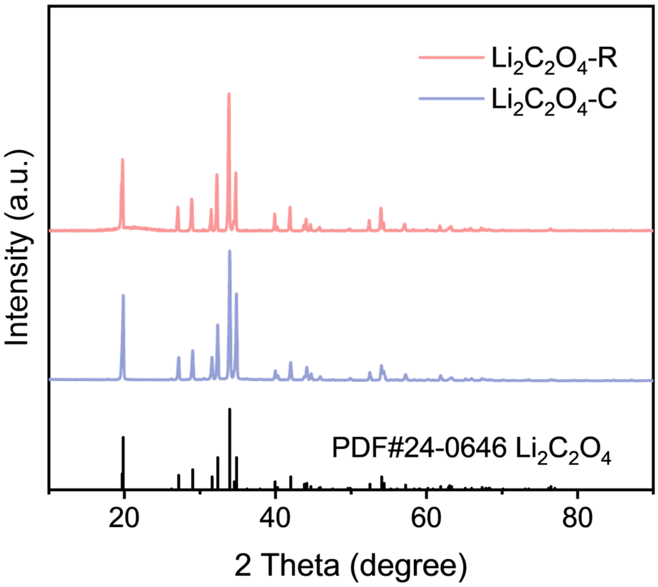

Figure S3. XRD patterns of commercial lithium oxalate ($Li_2C_2O_4$-C) and recrystallized lithium oxalate ($Li_2C_2O_4$-R).

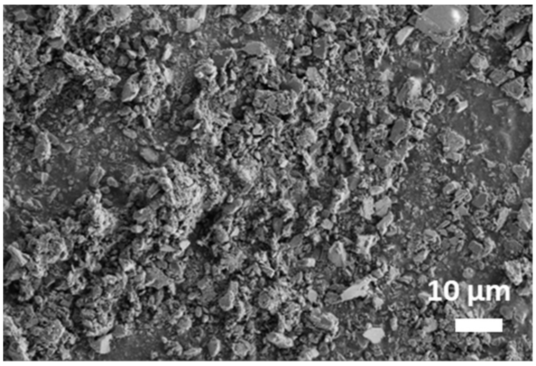

Figure S4. SEM image of recrystallized lithium oxalate ($Li_2C_2O_4$-R).

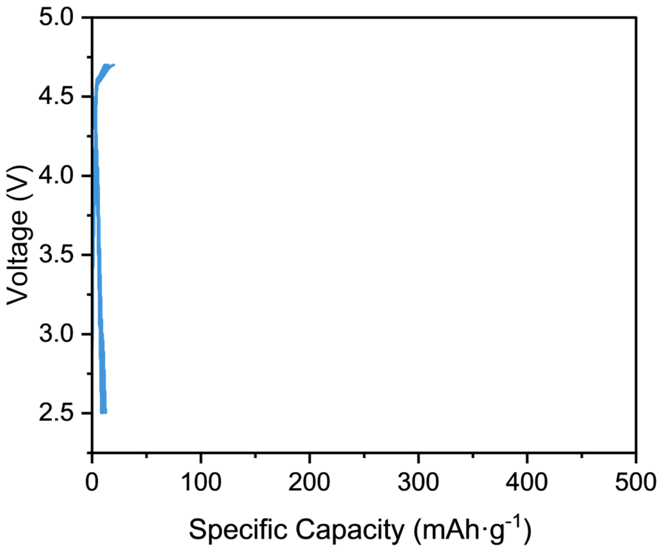

Figure S5. Charge/discharge curves of $Li_2C_2O_4$-R from the 2nd to 5th cycles.

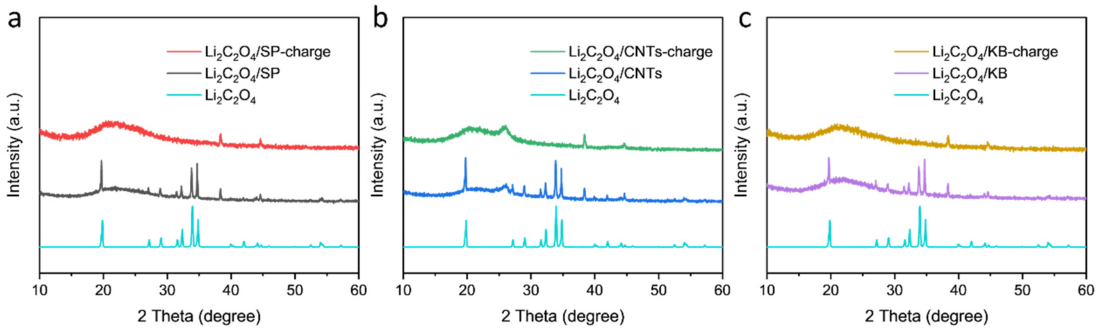

Figure S6. XRD patterns of $Li_2C_2O_4$ electrodes using different conductive agents before and after charging: (a) SP, (b) CNTs, (c) KB.

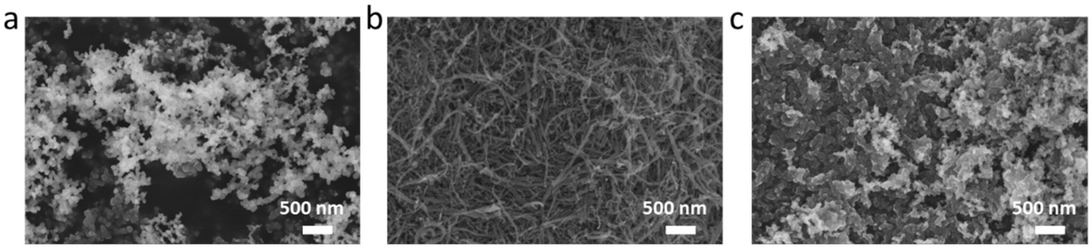

Figure S7. SEM images of (**a**) SP, (**b**) CNTs, (**c**) KB.

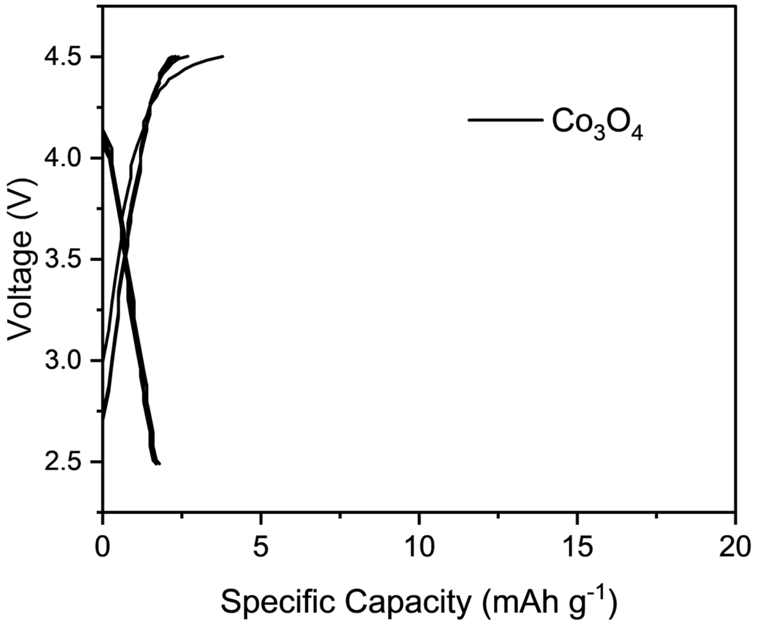

Figure S8. The five charge-discharge curves of Co_3O_4 ‖ Li half cell.

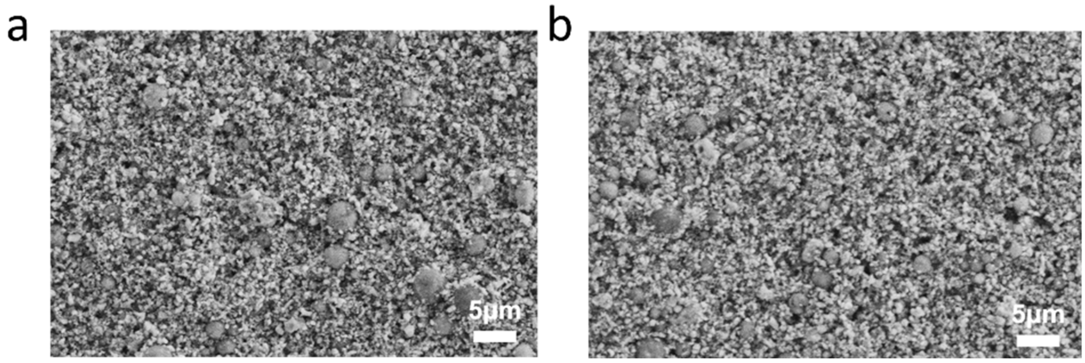

Figure S9. SEM images of the hybrid electrode of LiFePO4 and LCK80 (**a**) before and (**b**) after charging.

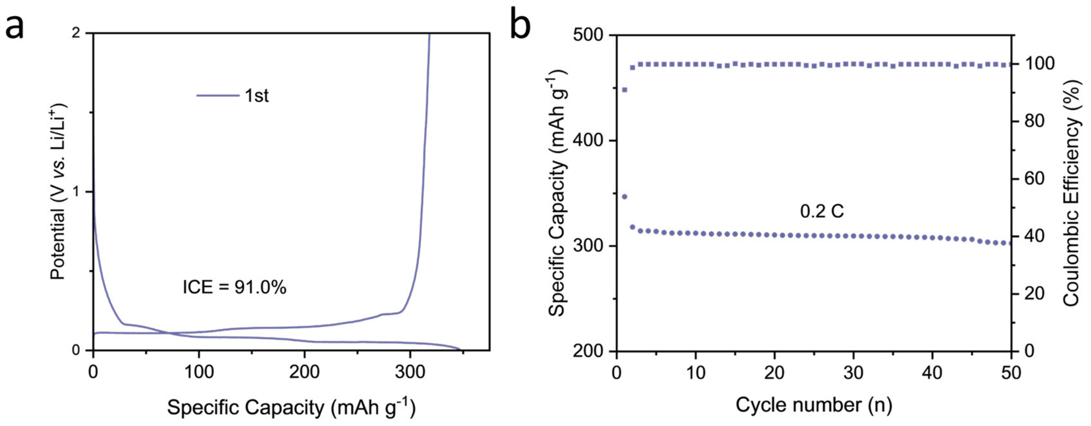

Figure S10. (**a**) Discharge/charge curve of graphite at first cycle at 0.1C. (**b**) Cycling performance of graphite at 0.2 C (1 C = 372 mAh·g^{-1}).

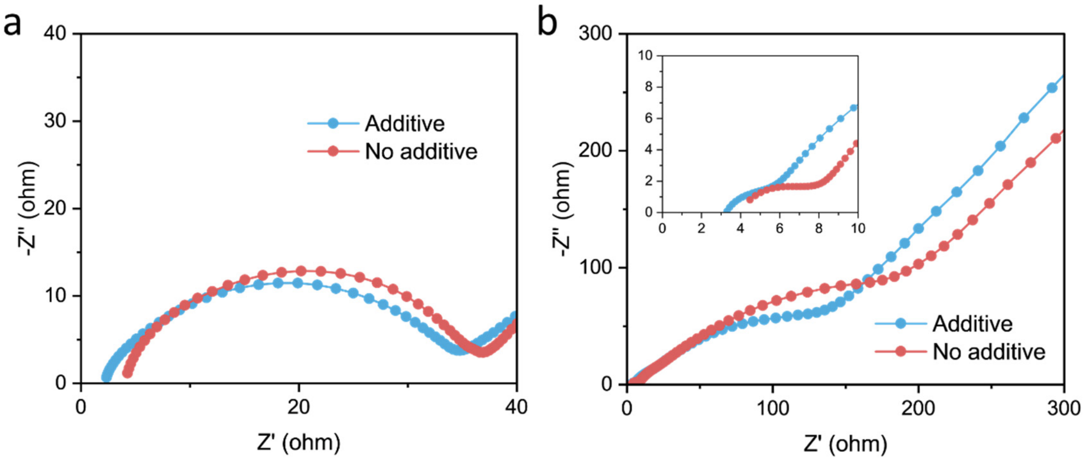

Figure S11. Electrochemical impedance spectroscopy of (a) LFP, (b) graphite after the first cycle.

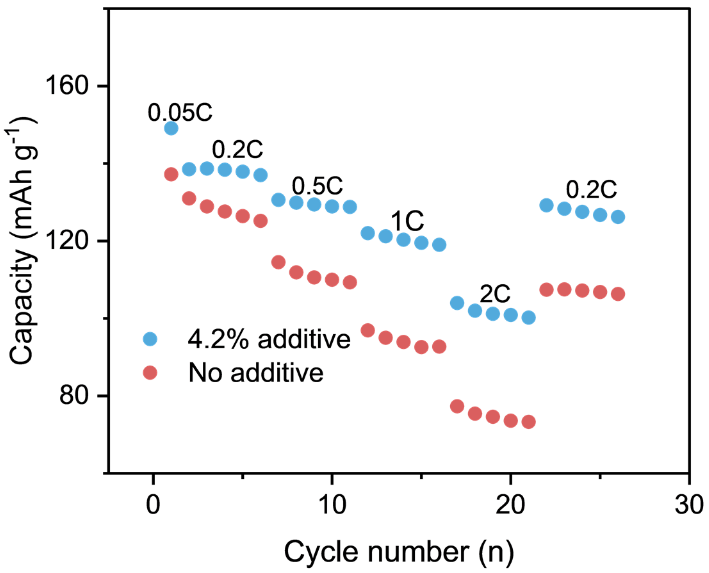

Figure S12. Rate performance of LFP‖graphite full cells.
